# Arthroscopic Bankart versus open Latarjet as a primary operative treatment for traumatic anteroinferior instability in young males: a randomised controlled trial with 2-year follow-up

**DOI:** 10.1136/bjsports-2021-104028

**Published:** 2021-09-22

**Authors:** Juha Kukkonen, Sami Elamo, Tapio Flinkkilä, Juha Paloneva, Miia Mäntysaari, Antti Joukainen, Janne Lehtinen, Vesa Lepola, Milja Holstila, Tommi Kauko, Ville Aarimaa, FINNISH (Finnish Instability Shoulder Study) Investigators, Anssi Ryösä

**Affiliations:** 1 Orthopaedics and Traumatology, Turku University Hospital, Turku, Finland; 2 Faculty of Medicine, University of Turku, Turku, Finland; 3 Surgery, Division of Orthopaedic and Trauma Surgery, Satakunta Central Hospital, Pori, Finland; 4 Surgery, Division of Orthopaedic and Trauma Surgery, Oulu University Hospital, Oulu, Finland; 5 Orthopaedics and Traumatology, Central Finland Central Hospital, Jyvaskyla, Finland; 6 Orthopaedics and Traumatology, Helsinki University Hospital, Helsinki, Finland; 7 Orthopaedics and Traumatology, Kuopio University Hospital, Kuopio, Finland; 8 Orthopaedic Unit, Tays Hatanpää Hospital, Tampere University Hospital, Tampere, Finland; 9 Pohjola Hospital Tampere, Tampere, Finland; 10 Department of Radiology, Turku University Hospital, Turku, Finland; 11 Auria Clinical Informatics, Turku University Hospital, Turku, Finland

**Keywords:** shoulder, sports medicine, trauma, upper extremity, orthopedics

## Abstract

**Objectives:**

To compare the success rates of arthroscopic Bankart and open Latarjet procedure in the treatment of traumatic shoulder instability in young males.

**Design:**

Multicentre randomised controlled trial.

**Setting:**

Orthopaedic departments in eight public hospitals in Finland.

**Participants:**

122 young males, mean age 21 years (range 16–25 years) with traumatic shoulder anteroinferior instability were randomised.

**Interventions:**

Arthroscopic Bankart (group B) or open Latarjet (group L) procedure.

**Main outcome measures:**

The primary outcome measure was the reported recurrence of instability, that is, dislocation at 2-year follow-up. The secondary outcome measures included clinical apprehension, sports activity level, the Western Ontario Shoulder Instability Index, the pain Visual Analogue Scale, the Oxford Shoulder Instability Score, the Constant Score and the Subjective Shoulder Value scores and the progression of osteoarthritic changes in plain films and MRI.

**Results:**

91 patients were available for analyses at 2-year follow-up (drop-out rate 25%). There were 10 (21%) patients with redislocations in group B and 1 (2%) in group L, p=0.006. One (9%) patient in group B and five (56%) patients in group L returned to their previous top level of competitive sports (p=0.004) at follow-up. There was no statistically significant between group differences in any of the other secondary outcome measures.

**Conclusions:**

Arthroscopic Bankart operation carries a significant risk for short-term postoperative redislocations compared with open Latarjet operation, in the treatment of traumatic anteroinferior instability in young males. Patients should be counselled accordingly before deciding the surgical treatment.

**Trial registration number:**

NCT01998048.

## Introduction

Anteroinferior shoulder dislocation is a frequent sports-related trauma,^1 2^ which is often accompanied by labral, ligament and even bony lesions of the glenohumeral joint. In up to 67% of cases, initial conservative treatment fails depending on the age and activity level of the patient. Thereafter, operative treatment may be advocated to address the resulting instability.^3^ Despite operative treatment, instability may recur particularly in young male patient populations,^4–6^ causing pain and discomfort, and potentially long-term wear of the glenohumeral joint.^7 8^

The Bankart procedure is the most commonly and widely used surgical intervention to treat shoulder instability.^9 10^ In this operation, the torn labrum and inferior glenohumeral ligament (IGHL) are anatomically reattached to the glenoid rim to re-establish the normal anatomy and stability of the joint.^11^ In addition, procedures may be carried out to address potential bony lesions, that is, the attachment of bony avulsions, or remplissage for a Hill-Sachs lesion.^12 13^ In spite of this, after a Bankart repair, dislocations may recur in up to 54% of cases in 10-year follow-up.^14^ Another potential treatment for treating shoulder instability is block bone procedures, which have been reported to be especially successful in the presence of glenoid bone deficiency.^15^ In recent years, Latarjet, a coracoid bone block procedure, has gained growing popularity. This procedure involves a non-anatomical transfer of the coracoid process and cojoined tendon to the glenoid neck.

Although Latarjet is reported to have a high success rate,^16–19^ studies have reported that patients who have undergone a previous arthroscopic Bankart repair before a Latarjet procedure are at risk of inferior outcomes.^20 21^ For this reason, Latarjet has been favoured as the primary procedure in shoulder anteroinferior instability. However, to date, no randomised controlled trial has compared the outcome of Bankart and Latarjet operations as the primary treatment for anteroinferior shoulder instability.

The aim of this trial was to compare the success rate of the arthroscopic Bankart procedure with the open Latarjet procedure. The hypothesis was that open Latarjet operations result in fewer redislocations, compared with arthroscopic Bankart in the primary surgical treatment of traumatic shoulder anteroinferior instability in young male populations.

### Patients and methods

This was a multicentre randomised controlled superiority trial carried out in eight public hospitals in Finland.

### Study population

**Figure 1:**
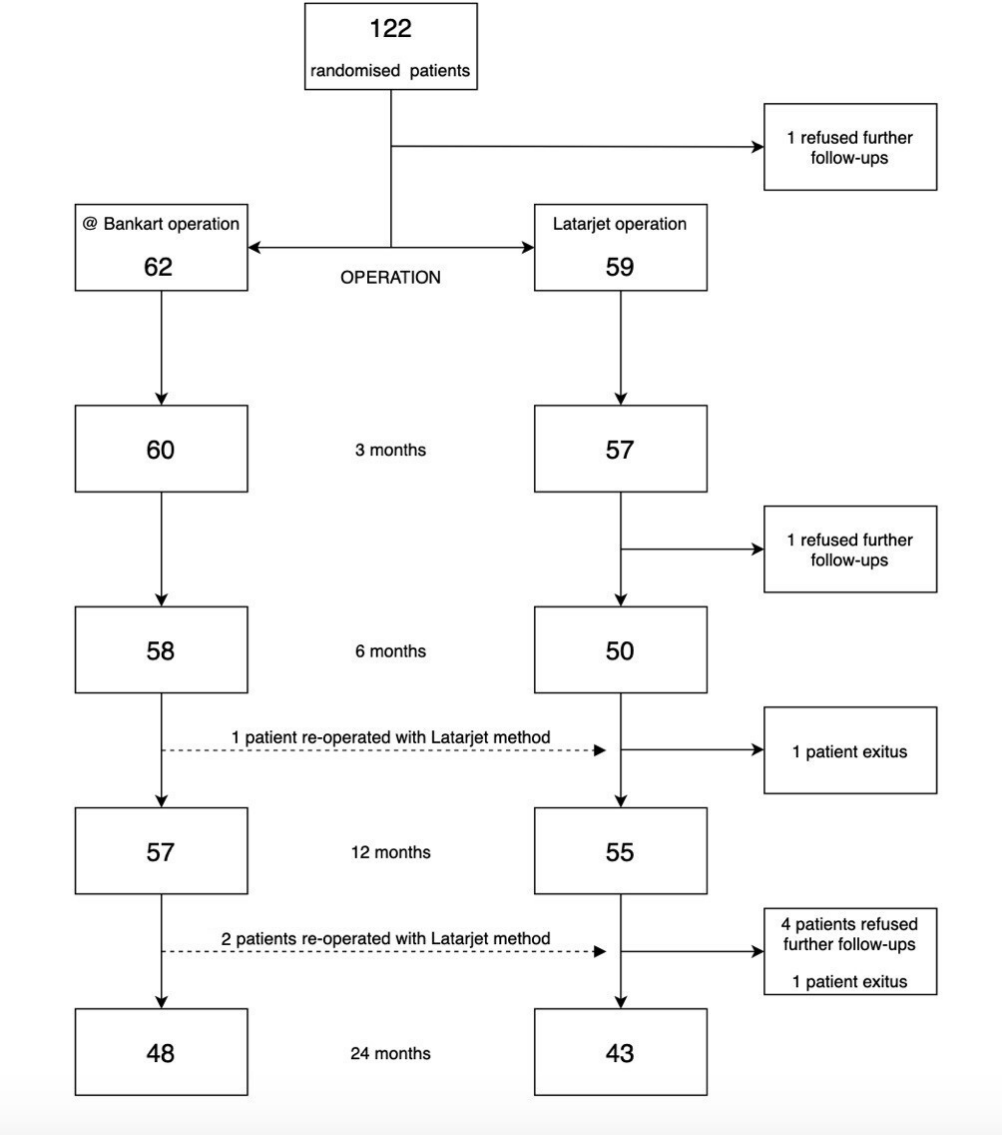
Flow chart of the study.

All young male patients, aged between 16 and 25 years, were screened for the trial if they had been referred to the participating institutes with anteroinferior shoulder instability after an initial traumatic dislocation. The inclusion and exclusion criteria are presented in box 1. The trial and the procedures were explained to all eligible patients, who were asked for a written consent. The enrolled patients were randomised into either the arthroscopic Bankart or the open Latarjet procedure (figure 1).

Box 1 **Inclusion and exclusion criteria**

**Criteria for inclusion**

- Involuntary redislocation, subluxation or fear of shoulder dislocation after a primarily conservatively treated traumatic anteroinferior shoulder dislocation.
- Clinically documented anteroinferior instability (ie, a positive apprehension and relocation test).
- Young adult male patient 16–25 years of age.

**Criteria for exclusion**

- Non-congruency of the glenohumeral joint on imaging investigations.
- Concomitant fractures requiring operative treatment of the humerus or the scapula (other than Hill-Sachs lesion or bony Bankart lesion).
- Severe grade 2 or above (Samilson and Prieto) osteoarthritis of the glenohumeral joint.
- Humeral avulsion of glenohumeral ligaments.
- Concomitant ipsilateral plexus or axillar nerve injury affecting motor function.
- Life threatening other concomitant injuries (ie, multitrauma patient).
- Stiffness of the glenohumeral joint.
- Intellectual disability, history of seizures with high risk of recurrence, existing significant malignant, haematological, endocrine, metabolic or rheumatoid disease.
- Previous ipsilateral shoulder surgery.
- History of alcoholism, drug abuse, psychological or other emotional problems that are likely to invalidate informed consent.
- Patient’s denial.

### Clinical assessment

The patients were assessed for clinical stability using the Jobe relocation test.^22^ Hyperlaxity was defined as external rotation above 90° and/or the Gagey hyperabduction test above 100°.^23^ The range of motion of the glenohumeral joint was measured using a goniometer. The participation and level of sports activities were recorded. Clinical scoring methods, supervised by a physiotherapist, included the Western Ontario Shoulder Instability Index (WOSI),^24^ the pain Visual Analogue Scale (VAS),^25^ the Oxford Shoulder Instability Score (OSIS),^26^ the Constant Score (CS) with subscores,^27^ the Subjective Shoulder Value (SSV)^28^ and the Instability Severity Index Score (ISIS).^4^

### Imaging

**Figure 2:**
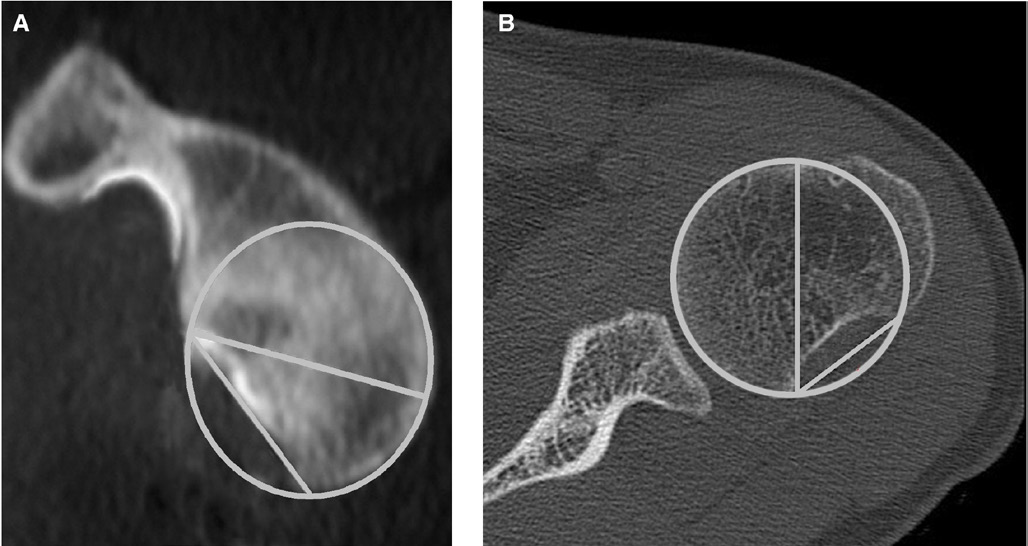
(A) The glenoid bone defect is considered significant when the tangential length of the defect is equal or more than 50% of the maximal width of the glenoid surface on a two-dimensional en face CT view.^29^ (B) The humeral defect is considered significant when the maximal width of the Hill-Sachs defect is equal or more than 40% of the diameter of the humeral head on a two-dimensional axial CT view.^30 31^

Preoperative plain films, CT and MRI were carried out. The CT images were assessed for significant bony deficiency of the glenoid. This was defined as the tangential length of the defect equal or more than 50% of the maximal width of the glenoid surface on a two-dimensional ‘en face’ CT view.^29^ For the humerus, it was defined as the width of the Hill-Sachs defect equal or more than 40% of the diameter of the humeral head on a two-dimensional axial CT view^30 31^ (figure 2).

### Operative treatment

All operations were carried out by experienced shoulder surgeons. Before commencing the trial, the surgeons held a wet-lab consensus meeting to decide how to perform both operations uniformly and in the best possible way.

### Bankart operation (group B)

The patient was prepared in a lateral decubitus or a beach chair position under general anaesthesia, supplemented with an interscalene block. The intra-articular findings were recorded and the anteroinferior labrum and the IGHL were mobilised until the subscapular muscle fibres were visible. The IGHL complex was then lifted and reattached anatomically to the freshened corner of the glenoid using two to three suture anchors, according to the surgeon’s preference, to recreate labral bumber and capsular tension. In cases of a significant Hill-Sachs defect, an additional remplissage procedure was performed, according to the surgeon’s decision, by inserting one to two more suture anchors into the deepest portion of the Hill-Sachs defect and tying the infraspinatus tendon down to fill the bony defect. The wounds were closed and the arm was placed in a sling for 3 weeks.

### Latarjet operation (group L)

The patient was prepared in a beach chair position under general anaesthesia supplemented with an interscalene block. A diagnostic arthroscopy was performed before the Latarjet operation. The intra-articular findings were recorded. In cases of a significant Hill-Sachs defect, an additional remplissage procedure was performed according to the surgeon’s decision by inserting one to two more suture anchors into the deepest portion of the Hill-Sachs defect and tying the infraspinatus tendon down to fill the bony defect. Thereafter, an open Latarjet operation was performed according to the standard techniques described by de Beer and Roberts or Young *et al* 32 33 using a deltopectoral approach by reattaching the coracoid process onto the freshened neck of the glenoid, just medial to the joint line with two screws and washers. The wounds were closed, and the arm was placed in a sling for 3 weeks.

All patients were invited to physiotherapy 3 weeks postoperatively. The physiotherapy first started with exercises involving a gradual range of motion, and progressed individually to active exercises during the first 6 weeks. All maximal force-requiring activities were restricted for the first 3 months and contact sports for 6 months.

In cases of recurrence of instability, the patient was individually assessed and, when necessary, reoperated on according to the preference of the physician.

### Outcome

The patients were clinically assessed and interviewed at 3 months, 6 months, 1 and 2 years postoperatively by a clinician or physiotherapist. The primary outcome measure of this trial was the patient reported recurrence of shoulder instability that is, glenohumeral dislocation at 2 years. The secondary outcome measures included clinical apprehension, sports activity level, absolute values in WOSI percentage, VAS, OSIS, CS and SSV scores. In addition, the progression of potential osteoarthritis was assessed from plain films according to Samilson and Prieto^34^ and from MRI images according to the modified osteoarthritis cartilage histopathology assessment system (Osteoarthritis Research Society International (OARSI)) grading by Pritzker *et al* 35 at 2-year repeat imaging.

### Patient involvement

There was no active patient involvement in the design, conduct, reporting or dissemination plans of the study.

### Power, randomisation and statistics

The power calculation was based on the assumed rate of redislocations: 10% in the Latarjet group and 35% in the Bankart group. It was expected that 90% of all redislocations would occur within the first 2 years. When the minimal significance (α) and statistical power (1 − β) were set at 0.05 and 0.80, respectively, the total number of patients needed per group was 43. In order to compensate for the possible drop-outs (estimate 15%), a total of 122 patients were recruited into the study. The randomisation took place 1 day prior to surgery with a bloc size of 6 and was stratified according to institute and the significant bony deficiency detected in the CT in either the glenoid or the humerus. Turku University Hospital served as the randomisation centre for the study.

Intention-to-treat (ITT) analysis was used for primary and secondary outcomes. The data were analysed using methods suitable for clinical trials regarding the comparison of parallel treatment groups with repeated measurements. The primary technique was the analysis of variance of repeated measurements together with generalised linear mixed models for longitudinal data. The Kaplan-Meier method and Cox regression models were used to calculate and illustrate the risk of redislocation. These analytical tools cover methods for analysing different kinds of outcome variables, and are applicable although there is missing data in the measurements during the follow-up. The primary statistical software was the latest release of SAS system 9.4 for windows, SAS Institute.

## Results

The baseline patient demographic data are presented in table 1. At 2-year follow-up, 91 patients were available for analyses (drop-out rate 25%). The mean age of the patients at the time of the operation was 21 years (SD 2.7) in both groups. Preoperatively, in groups B and L, respectively, there were 19 and 18 significant bony defects of the glenoid, and 19 and 18 of the humerus, in the CT analysis. The mean ISIS score was 2.8 (SD 1.7) and 2.7 (SD 1.9), and the median number of dislocations before surgery were 6 in group B and 6 in group L.

**Table 1:** Demographics of the participants allocated to Bankart or Latarjet procedure

Group	Bankart	Latarjet
N	62	59
Mean age (SD) (range)	21.4 (2.7) (16–25)	21.4 (2.7) (16–25)
Mean weight, kg (SD) (range)	78.3 (12.6) (54–125)	79.5 (12.7) (59–113)
Side (right/left)	21/41	28/30
Hyperlaxity (n)	11	8
History of contact sports (n)	27	25
Mean Instability Severity Index score (range)	2.8 (0–6)	2.7 (0–9)
Significant Hill-Sachs lesion in CT (n)	19	18
Significant bony Bankart lesion in CT (n)	19	18

In the arthroscopic assessment, the cartilage was recorded as normal on the glenoid side in all patients and as frayed on the humeral side in three patients from group B and two patients from group L. Furthermore, there was one clearly engaging Hill-Sachs lesion in both groups. Remplissage was performed in 15 patients from group B and 3 patients from group L (8/19 and 2/18 patients with preoperatively evaluated significant humerus bone defect), respectively.

**Figure 3:**
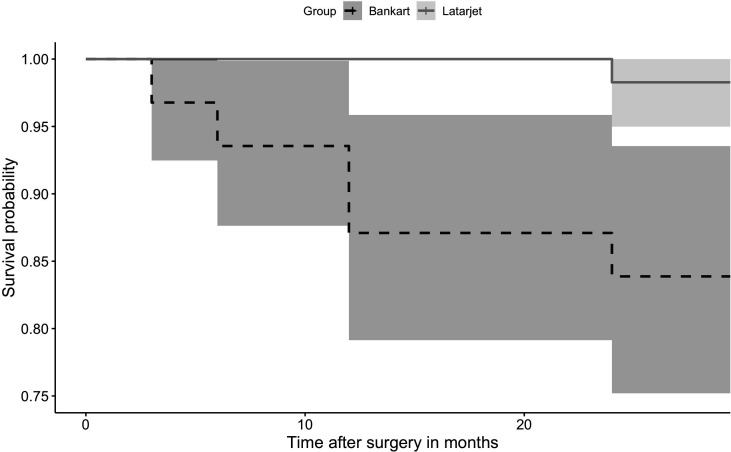
Kaplan-Meier survival graph with 95% CIs (shaded area) of the two treatment groups with redislocation as an endpoint.

There were 10 patients with redislocations in group B (21%) and 1 in group L (2%), p=0.006. Three patients with redislocations in group B were subsequently reoperated on using an open Latarjet method. The between group survival analysis regarding redislocations is presented in figure 3. The HRs (with 95% CI) for early redislocation in group B in case of hyperlaxity, involvement in contact sports, significant humeral and glenoid defects were 0.53 (0.11 to 8.21), 0.48 (0.15 to 2.72), 0.21 (0.06 to 2.18) and 1.51 (0.49 to 7.81), respectively.

**Figure 4:**
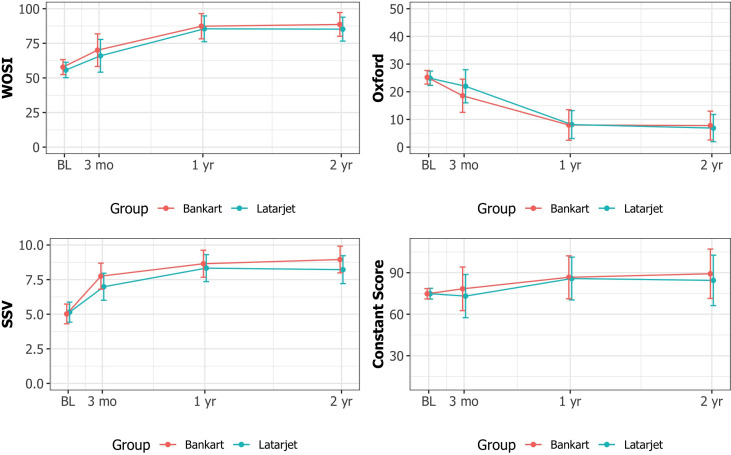
Mean outcome scores with 95% CI whiskers. (Western Ontario Shoulder Instability (WOSI), Oxford Shoulder Instability Score (Oxford), Subjective Shoulder Value (SSV) and Constant Score). BL, baseline.

The mean preoperative WOSI was 57.8 (SD 20.3) in group B and 55.7 (SD 20.7) in group L. At 2-year follow-up, the mean WOSI was 88.4 (SD 10.1) and 85.4 (SD 12.3), respectively p=0.201. A total of 84 patients fully completed all of the clinical follow-up scores, and there was no statistical significance between group differences in the scores. The outcome scores are presented in figure 4. There were 16 (33%) and 7 (16%) patients with signs of clinically positive apprehension (p=0.157) in groups B and L, and 1 (9%) and 5 (56%) patients who had returned to their previous top level of competitive sports (p=0.004), at follow-up, respectively.

**Figure 5:**
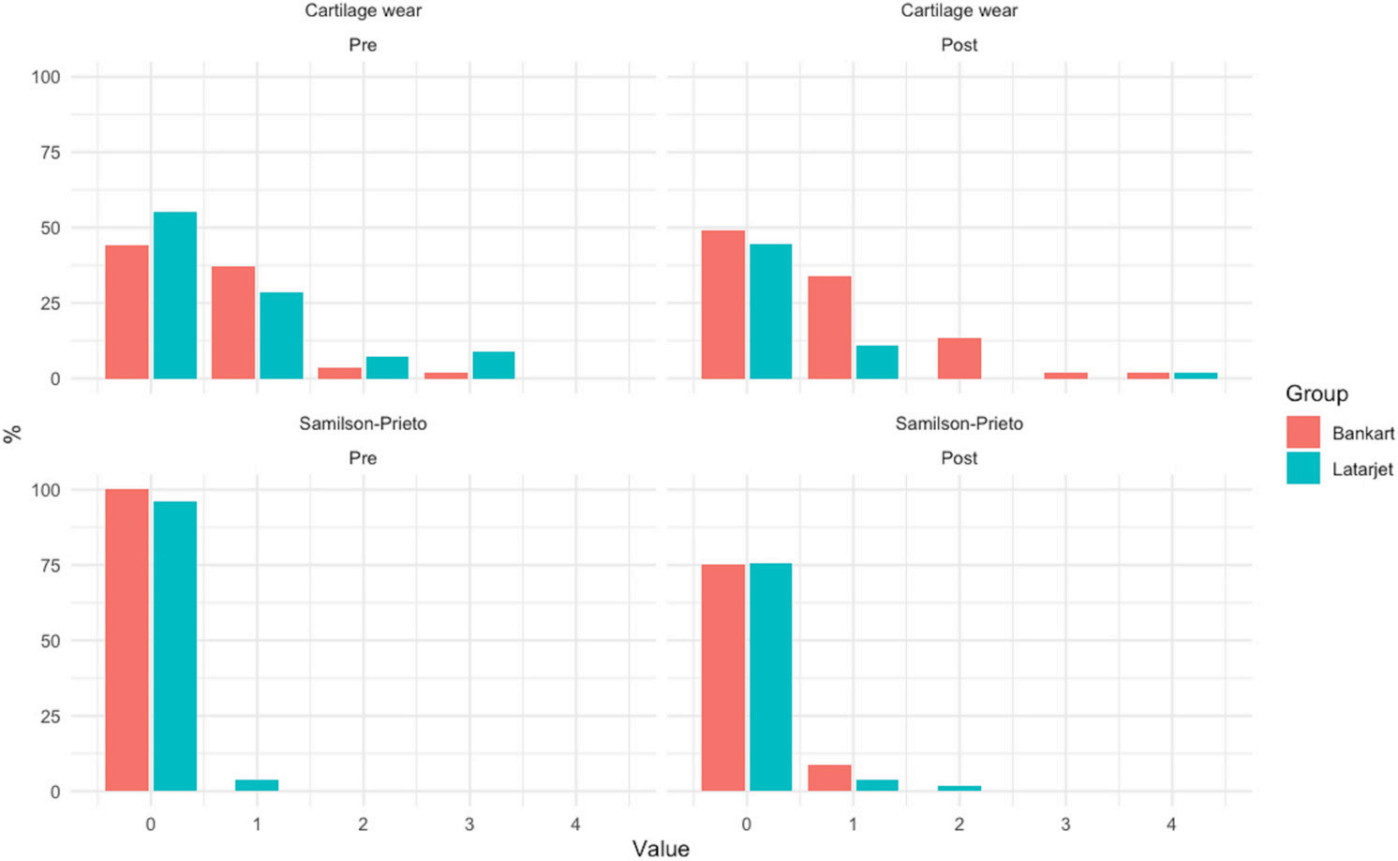
Detected degenerative osteoarthritic changes preoperatively (left column) and at 2-year (right column) follow-up in MRI (upper row) and plain film (lower row) imaging. MRI cartilage wear grade 0: normal, grade 1: focal areas of hyperintensity, grade 2: cartilage fissures: grade 3: focal cartilage ulcerations, grade 4: full thickness cartilage loss (modified OARSI grading by Pritzker *et al*). Samilson-Prieto grade 0: normal, grade 1: osteophyte <3 mm, grade 2: 3–7 mm, grade 3: >7 mm. OARSI, Osteoarthritis Research Society International.

In repeat plain film and MRI analyses, there was no statistically significant progression of glenohumeral joint degenerative osteoarthritic changes in either group when compared with the preoperative state (figure 5).

No treatment-related complications occurred in either group.

## Discussion

The main result of this trial was that the arthroscopic Bankart operation associated more significantly with redislocations and the need for subsequent revision surgery compared with the open Latarjet operation. The Latarjet operation was associated with higher rate of returning to previous top level of competitive sports.

Despite the significant difference between groups, the number of early redislocators in the Bankart group at 2 years was low compared with previously reported long-term follow-up studies.^8 36^ This may be partly due to meticulous surgical technique and the possibility for an additional remplissage procedure. Nevertheless, the 2-year follow-up is likely to be too short to catch all of the redislocators in the two groups. Additional redislocators can be expected to merge especially in the Bankart operated group at later follow ups in our trial.^14 17^ Although many patients had an initial bony lesion, the number of significant bone deficiencies was proportionally low which represented a potential advantage for the Bankart operation. At some clinical practices, significant bony lesions could be considered a contraindication for Bankart repair. An additional remplissage was received by 42% of patients in the Bankart group who had significant Hill-Sachs lesions in preoperative evaluation. In contrast, only 11% of patients with a similar lesion received remplissage in the Latarjet group. We assume that the surgeons did not consider that remplissage was necessary as often in conjunction with Latarjet as with Bankart. Furthermore, it can be argued that the two-dimensional size of the bone defect itself is an insufficient measure of clinically significant bone insufficiency. It has been previously reported that the amount of bone loss is associated with the number of dislocation episodes.^37 38^ This highlights the importance of surgically addressing this disorder early. Interestingly the ISIS scoring nor its parameters were not significantly associated with poor early outcome in our trial, contrary to previous reports.^4 39 40^

Despite redislocation, the patient-reported outcome was similar in both groups. This may be interpreted as the sudden manifestation of redislocation without preceding symptoms. On the other hand, the high and extreme demands of functionality, may not be sufficiently captured by the questionnaires as suggested by the higher percentage of patients returning to a competitive level of sports in the Latarjet group in our trial. Furthermore, the results were analysed according to ITT, and the open Latarjet revision procedure in three patients may have somewhat compensated for the otherwise potentially worse outcome in the Bankart group.

Both of the operative methods that were compared are old, Bankart was described in 1923^41^ and Latarjet in 1954.^42^ Despite their long history, very little high-quality comparative evidence has been put forth so far. To our knowledge, this is the first randomised controlled trial on this topic. In a systematic review by Rollick *et al*, the estimated redislocation rate was 15.1% following arthroscopic Bankart repair compared with 2.7% after Latarjet repair.^43^ In a systematic review and a meta-analysis of 795 shoulders, the Latarjet procedure conferred a significantly lower risk of recurrence and redislocation compared with the Bankart procedure—recurrence with the Bankart repair was approximately twofold higher.^16^ Our results are in accordance with these earlier reports. The technical success of operative treatment in our trial is characterised by no early severe complications. However, we did not record the possible postoperative transient stiffness in this trial. In previous reports, the Bankart operation has been associated with lower complication rates compared with the Latarjet operation.^44^ This must be kept in mind when counselling patients. It is also noteworthy that there was a slight, although statistically insignificant, progression of cartilage wear at 2-year follow-up in both groups. The consequence of this finding requires a further follow-up of these patients.

There are certain limitations in this trial. First, one procedure was performed arthroscopically and the other used an open approach. The patients and treatment team were openly aware of the treatment allocation, and although both treatments were regarded as routine practice, we do not know if this affected the outcome. Despite reports on the non-significance between open and arthroscopic approaches,^45 46^ there are also contradictory findings.^47^ Second, a relatively short follow-up may be considered another weakness of this trial. It may be that instability recurs at later sporadic time points, and also that degeneration of the glenohumeral joint progresses. This might occur non-synchronously between the groups. Therefore, these results must be interpreted as preliminary. Third, the exact number of redislocations in each patient with treatment failure is not known. However, the reoperations, reflecting a repetitive problem, were carefully recorded. Fourthly, young males are an especially demanding group of patients with low compliancy, and accordingly, we experienced a moderate rate of drop outs in our trial. To some extent, this also emphasises the need for operational success and long-standing treatment effect in their case. Finally, this trial excluded patients who were female, older than 25 years and who had experienced an atraumatic onset of symptoms. These patients may behave differently, and therefore, the findings of this trial are not applicable to all patients with shoulder instability.

## Conclusions

The arthroscopic Bankart operation carries a significant risk for short-term postoperative redislocations and a need for additional surgery compared with the open Latarjet operation, in the treatment of traumatic anteroinferior instability in young males. In this patient population, returning rate to previous top level of competitive sports was higher after the Latarjet operation compared with the Bankart procedure. Patients should be counselled accordingly before deciding on surgical treatment. The short-term patient-reported outcomes are similar in both the Bankart and the Latarjet procedures. However, further studies are needed to evaluate the long-term comparative clinical and radiological outcome of these two procedures.

**What are the findings?**

- The arthroscopic Bankart procedure was associated with an increased risk of shoulder redislocations compared with the Latarjet procedure in young males with shoulder traumatic anteroinferior instability.

**How might it impact on clinical practice in the future?**

- The Latarjet procedure may be the preferred operative treatment option for traumatic anteroinferior shoulder instability in young males.

10.1136/bjsports-2021-104028.supp1 Supplementary data

## Data Availability

Data are available on reasonable request.
