# Supplementary material for: Arthroscopic Bankart versus open Latarjet as a primary operative treatment for traumatic anteroinferior instability in young males: a randomised controlled trial with 2-year follow-up
Source: Br J Sports Med. 2021 Sep 22;56(6):327–32. doi: 10.1136/bjsports-2021-104028 (PMC8899479; doi:10.1136/bjsports-2021-104028)
Supplement: Supplementary data [file bjsports-2021-104028supp001.pdf]

August 8th 2013

investigation plan

Operative treatment of traumatic anteroinferior shoulder instability in young male patients

The outcome of arthroscopic Bankart vs. open Latarjet stabilization surgery, a randomized controlled trial

Principal investigator

MD PhD Ville Äärimaa

Co-investigators:
MD M.Sc.(Tech) Sami Elamo
Docent Tapio Flinkkilä
MD PhD Antti Joukainen
MD Juha Kukkonen
Docent Janne Lehtinen
MD PhD Vesa Lepola
MD PhD Mika Paavola
MD PhD Juha Paloneva
MD PhD Esa Tuominen

Address of correspondence:
Ville Äärimaa, MD, PhD
Artro-process
Department of Orthopaedics and Traumatology
Turku University Hospital
TYKS kirurginen sairaala
Luolavuorentie 2
PL 28
20701 Turku
Tel.+358 (0)2 313 0000
email: ville.aarimaa@utu.fi

1) INTRODUCTION

Glenohumeral joint is the most mobile joint in the human body. Its bony architecture allows a wide and nearly circumferential range of motion. As a consequence of unrestricted motion, the glenohumeral joint is prone to instability, i.e. the humeral head may dislocate off the scapular glenoid plate especially in the anteroinferior direction (Rockwood *et al.* 2009, Provencher and Romeo 2012). The most common mechanism of shoulder dislocation is a fall or impact on abducted and externally rotated arm. This is typically a sports related injury and affects especially young males (Lievaag 2011). A primary anteroinferior shoulder dislocation nearly always disrupts the anterior labrum and the anterior inferior glenohumeral ligament (IGHL complex) (Perthes-Bankart laesion), which is considered the primary passive soft-tissue restraint of the glenohumeral joint. There may also be other frequent accompanying laesions, such as an impression fracture of the humeral head (Hill-Sachs), or of the anterior glenoid rim (bony Bankart). The plain x-rays may underestimate this bony pathology in the glenohumeral joint and a computed tomography imaging allows a superior and more careful investigation of the bony morphology. On the other hand magnetic resonance imaging helps to detect the soft tissue injuries. The disruption of passive shoulder restraints and bony architecture may lead to further shoulder instability after the first time dislocation. Among patients below 25 years of age redislocations and residual instability occur in up to 50% of cases after primary dislocation (Hovelius 2008).

The most common operation to treat shoulder instability is arthroscopic capsulolabral repair (Bankart operation). The torn labrum and IGHL are re-attached to the glenoid rim with suture anchors. The results of arthroscopic Bankart repairs are regarded similar to those of open Bankart repairs (Hobby *et al.* 2007). Recurrence rates of instability after a Bankart repair have been variable ranging from 5 % to 35% in 2 to 10 years' follow-up. Risk factors for failure after arthroscopic Bankart repair have included young age, bony lesions either on humeral or glenoid side, increased laxity of joints (hyperlaxity) and contact sports. Balg and Boileau (2007) found that in young patients under 20 years of age, with glenoid and/or humeral bone defects, and who participated in competitive contact or overhead sports, there is a 70 % failure rate after arthroscopic stabilisation surgery. A follow-up study in Oulu University Hospital recognized young age as the most important risk factor for failure after arthroscopic Bankart repair. If the patient's age at the time of surgery was under 20 years, the failure rate was 44% after arthroscopic Bankart repair during five years follow-

up. (Flinkkilä *et al.* 2010). Accordingly, Castagna *et al.* (2012) reported a 21% failure rate in 13−18 year-old patients who participated in contact sports. Boileau *et al.* (2006) reported that 70% of recurrences of instability symptoms take place within two years after the operative intervention. In a previous study from Oulu University Hospital, 75% of the failures took place within two years and 96% within three years after arthroscopic Bankart operation (Flinkkilä *et al.* 2010).

More robust operative techniques have evolved to achieve greater stability of the glenohumeral joint against eg. sports related recurrences (Roberts *et al.* 1999). In 1954 Michel Latarjet described an operation in which the coracoid process of the scapulae together with the conjoined tendon (short head of the biceps and coracobrachialis tendon) is detached, transferred through the subscapularis muscle and re-attached to the neck of the glenoid (Latarjet 1954). The stabilizing mechanism of this operation has been reported to be a bone block effect together with a sling effect of the conjoined tendon and inferior part of the subscapularis muscle. The Latarjet operation has gained popularity and is especially advocated in cases with bone defects on the glenoid side. The Latarjet operation (or its modification Bristow-Latarjet) has been reported to yield in high success rates (Matthes 2007). However, there are no studies with comparable groups of patient with Bankart and Latarjet operation. Many orthopaedic surgeons have doubts over Latarjet technique because of its nonanatomic nature. However, in a series by Hovelius et al., the Latarjet operation did not have any more negative long-term effects than open Bankart operation (Hovelius 2011). Only few studies have reported failures after Latarjet–operations and the rates of recurrence of instability have ranged from 0 to 10% (Allain *et al.* 1998, Hovelius *et al.* 2004).

Every episode of shoulder instability is likely to induce injury to the joint cartilage and further predispose to osteoarthritis within the glenohumeral joint. It has been reported that there are radiographically visible osteoarthritic changes in at least 20% of the patients 10 years after the primary shoulder dislocation injury (Hovelius 1996). Therefore, surgical intervention, ie. stabilization of the joint to avoid further dislocation episodes, may potentially protect the joint cartilage. However, the surgical treatment may also contribute to the development of osteoarthritis by inducing iatrogenic injury to the joint. Post-traumatic osteoarthritis of the glenohumeral joint may lead to increased disability and morbidity, and should be prevented if possible.

Kukkonen J, *et al. Br J Sports Med* 2022; 56:327–332. doi: 10.1136/bjsports-2021-104028

There are no previous prospective randomized studies investigating the difference in outcome between a Bankart and a Latarjet operation.

2) AIMS OF THE INVESTIGATION

1) Study the difference in outcome after arthroscopic Bankart operation compared with open Latarjet operation in the treatment of a residual instability after a traumatic primary dislocation in young males.
   Hypothesis: Open Latarjet operation yields in a significantly lower recurrence rate and a better subjective result.
2) Study the accuracy of plain x-rays on detecting bony laesions of the glenohumeral joint (Hill-Sachs and bony Bankart) compared to computed tomography imaging.
   Hypothesis: Plain X-rays give an inaccurate estimate of the bony lesions.
3) Study the development of osteoarthritic changes within the glenohumeral joint in the study population in 2 to 20 year time interval.
   Hypothesis: The osteoarthritic changes are related to the severity of primary injury and bone lesions.

3) PATIENTS AND METHODS

This randomized controlled study with parallel treatment groups will be conducted according to the revised Declaration of Helsinki by The World Medical Association and the ICH-guidelines for good clinical trial practice. The study will be submitted for approval to the Ethics Committee of the Hospital District of Varsinais-Suomi, Finland. The study will be conducted as a multicentre study in Finland at Turku University Hospital, Helsinki University Hospital, Tampere University Hospital / Tampere Hatanpää hospital, Jyväskylä Central Finland Hospital, Kuopio University Hospital, Oulu University Hospital and Satakunta Central Hospital Pori . Additional recruiting hospitals and researchers maybe included during the trial in case of difficulties in patient recruitment (less than 30 patients during the first 6 months). The study protocol will be registered at clinicaltrials.gov.

Kukkonen J, *et al. Br J Sports Med* 2022; 56:327–332. doi: 10.1136/bjsports-2021-104028

Patients with post-traumatic anteroinferior shoulder instability are referred to the participating institutes by local health care providers. All referred patients are called in for clinical and radiological examination by the investigators. If the patient is eligible and willing to participate in in the study a written informed consent is obtained. Patients who are eligible, but not willing to participate in the study, are asked for a permission to use their patient records for study purposes.

The recruited patients are informed of study protocol and that a stabilizing shoulder operation will be performed accordingly. The operation is scheduled within 6 weeks after recruitment. Right before the operation the patients are randomized in one of two studied treatment modalities.

Criteria for inclusion:

1. Subluxation or fear of shoulder dislocation after a previous, reduced and primarily conservatively treated (for more than 3 months) traumatic anteroinferior shoulder dislocation.
2. Redislocation after a primary shoulder dislocation.
3. Clinically documented anteroinferior instability (ie. a positive apprehension and relocation test (Jobe)).
4. X-ray (true ap, 30 degrees oblique ap, Y- and axillary projections), 2- and 3-dimensional computed tomography (2D and 3D CT) and magnetic resonance imaging arthrography (MRA) documentation of the joint.
5. Congruency of the shoulder joint on imaging investigations.
6. Young adult male patient 16-25 years of age (15 years < patient ≤ 26 years).
7. Patients willingness for operative treatment.
8. Written informed consent from participating subject.

Criteria for exclusion:

1. Non-congruency of the glenohumeral joint on imaging investigations.
2. Concomitant dislocated fractures (requiring operative treatment) of the humerus or the scapula (other than Hill-Sachs laesion or bony Bankart Laesion)
3. Severe grade 2 or above (Samilson et Prieto) osteoarthrosis of the glenohumeral joint detected in X-ray investigation.

Kukkonen J, *et al. Br J Sports Med* 2022; 56:327–332. doi: 10.1136/bjsports-2021-104028

4. A humeral avulsion of glenohumeral ligaments (HAGL) detected in MRA investigation.
5. Concomitant ipsilateral plexus or axillar nerve injury affecting motorical function.
6. Life threatening other concomitant injuries (i.e. multitrauma patient).
7. Stiffness of the glenohumeral joint (restricted passive external rotation less than 30 degrees measured in standing position, arm at side).
8. Age under 16 or above 25 years.
9. Open physis with significant growth expectation.
10. Intellectual disability, history of seizures with high risk of recurrence, existing significant malignant, haematological, endocrine, metabolic, or rheumatoid disease.
11. Previous ipsilateral shoulder surgery.
12. Cytostatic or corticosteroid medication.
13. History of alcoholism, drug abuse, psychological or other emotional problems that are likely to invalidate informed consent.
14. Disability to co-operate.
15. Patient's denial.

Randomization:

The randomization is divided into two main lists (each containing four separate lists for each risk profile) according to participating hospitals to keep the groups better balanced between the institutions (main list 1 = Turku, Helsinki, Pori and Tampere institutes, and main list 2 = Kuopio, Jyväskylä and Oulu institutes). Turku University Hospital serves as a randomization center for the study. A stratified randomization is used to control the equal distribution of two major previously reported risk factors for failure between the groups. There are thus 4 possible combinations of risk factors (0+0, 1+0, 0+1, 1+1), and within each risk profile block randomization with randomly selected block sizes of 2 to 4 is used. SAS statistical software is used to generate the randomization lists up to 60 patients for each risk profile within each main list. After informed consent the individual study center will contact Turku study nurse by phone and informs about the eligible patient and the scheduled operation day, and whether there is: 1) a significant glenoid bone defect/ bony Bankart-lesion or not, and 2) a significant humeral impression (Hill-Sachs) or not, according to APPENDIX 1. The study nurse will then activate the electronic follow-up registry for the patient. At two weeks before the planned operation the pre-operative measurements are performed by a

Kukkonen J, *et al. Br J Sports Med* 2022; 56:327–332. doi: 10.1136/bjsports-2021-104028

physiotherapist according to APPENDIX 7. The individual study center will then contact the study nurse who then performs and controls the randomization and informs the clinical investigator of the study group 2 days before the planned operation (on Friday if the operation is planned to take place on Monday) by sending an email and SMS-message to the operating surgeon and entering the randomized treatment group into the electronic patient registry. The clinical investigators are blinded from the randomization procedure. Recruitment and randomization is continued until 120 patients are enrolled in the study.

Following anamnestic patient characteristics are recorded:
age at primary injury, mechanism of primary injury, number of dislocation episodes, degree of instability (dislocations at sleep, during daily activities, at sports, no dislocations), level (elite, competitive, recreational, none) and intensity (times per week) to perform sports activities (type of sport), pain at rest, subjective visual analogue estimation of the shoulder condition, smoking habits, other diseases, type and ability to work, handedness, and subjective satisfaction on the treatment outcome on a 5-point Likert scale.

Following clinical patient characteristics are recorded:
clinical glenohumeral stability (jerk test, apprehension and relocation tests), clinical signs of hyperlaxity (external rotation range over 90° in 0° abduction position, and/or positive sulcus sign in the inferior drawer test, and/or a positive Gagey test (over 100°), and/or significantly increased translation in the anteroposterior drawer test).

Following shoulder scores are recorded:
Constant score, Western Ontario Shoulder Instability index (WOSI), Oxford Instability Score (Oxford score), Subjective Shoulder Value (SSV) and instability severity index score (ISIS). (Constant and Murley 1987, Kirkley *et al.* 1998, Balg and Boileau 2007, Gilbart and Gerber 2007). A blinded physiotherapist performs all scorings with patient wearing a shirt (covering the scars around the shoulder).
APPENDIX 2-5.

Following imaging investigation is performed:

Kukkonen J, *et al. Br J Sports Med* 2022; 56:327–332. doi: 10.1136/bjsports-2021-104028

X-ray (true ap, 30 degrees oblique ap, Y-, and axillary projections) APPENDIX 6, 2D and 3D CT, and MRA. All the imaging data will be sent and stored in the Turku University Hospital's picture archive PACS. Computed tomography will be taken of both shoulders (affected and unaffected side).

The timing and method of the data gathering is presented in APPENDIX 7.

Treatment groups:

1) Bankart operation

An arthroscopic Bankart operation is performed in general anaesthesia according to current practise (Provencher 2010). The intra-articular findings are recorded and the anteroinferior labrum and the IGHL are mobilized until subscapular muscle fibers can be seen. The IGHL complex is then re-attached to the freshened neck of the glenoid with 2 to 3 suture anchors according to surgeons preference to re-create labral bumber and capsular tension. In case of a significant Hill-Sachs defect an additional remplisage procedure may be performed according to surgeons' decision by inserting 1 to 2 more suture anchors, according to surgeon's preference into the deepest portion of the Hill-Sachs defect and tying the infraspinatus tendon down to fill the bony defect.

2) Latarjet operation

A diagnostic arthroscopy is performed before the Latarjet operation in general anaesthesia. The intra-articular findings are recorded and in case of a significant Hill-Sachs defect an additional remplisage procedure may be performed according to surgeons' decision by inserting 1 to 2 more suture anchors according to surgeon's preference into the deepest portion of the Hill-Sachs defect and tying the infraspinatus tendon down to fill the bony defect. Thereafter an open Latarjet operation is performed using standard techniques described by Walch or de Beer (de Beer *et al.* 2010, Young and Walch 2012). A deltopectoral incision is used. The coracoid process is osteotomized and, depending on the technique used, the ventral (Walch) or medial side (de Beer concruent arc modification) is prepared to bleeding bone. The coracoid process is then transferred through the middle of the subscapularis and re-attached on to the freshened neck of the glenoid, just medial to the joint line with two screws and washers, according to the surgeon's preference.

Kukkonen J, *et al. Br J Sports Med* 2022; 56:327–332. doi: 10.1136/bjsports-2021-104028

Postoperatively all the patients receive an 8-week sick leave and recipe of painkillers (paracetamol + codein 1x4 and ibuprofein 600mg 1x3) to be used if needed. The patient's arm is immobilized with a simple sling for three weeks. At three weeks the patient is called for physiotherapy and the patient is given guidance to painless free passive range of motion exercises. At six weeks active strengthening program is begun. Thereafter patient is asked to report any incidents regarding the studied shoulder by phone to study nurse. Participation in contact sports is forbidden for all patients until six months after the operative treatment. The patient is called in for a doctor's control visit at three months, six months, one year, two years, five years, ten years and twenty years postoperatively. In case of re-dislocation or signs of instability occurs the patient is called for and urgent clinical examination. If symptomatic and clinically documented residual instability exists the treatment is considered a failure and the further treatment is planned individually.

4) ETHICS AND SAFETY

This study is conducted according to ethics principles of Helsinki declaration. Both treatments used in this study are well established and considered safe, and used in current clinical practise. All observed or volunteered adverse events regardless of suspected relationship to the study will be recorded. The investigator assesses the likelihood of the adverse event to be caused by the study treatment on a six-grade causality scale (none, unlikely, possible, probable, definite, cannot be classified).

The patients are urged to report any adverse events immediately after appearance to the research nurse and research doctor (SE). The severity of all adverse events is assessed on a three-grade scale (mild, moderate, severe). All adverse events are dealt with in a symptomatically adequate manner and the patients are hospitalized if needed.

A blinded interim (coded groups) analysis of the available outcome data is performed after 60 patients have been recruited and operated on to confirm safety and ethical considerations of the study. In case either technique results in unacceptably high rate of recurrence of instability or other complications, premature discontinuation of the study is considered.

Kukkonen J, *et al. Br J Sports Med* 2022; 56:327–332. doi: 10.1136/bjsports-2021-104028

Ethics committee will be informed in case of study discontinuation.

5) PREMATURE DISCONTINUATION

A patient may withdraw consent and discontinue the study prematurely at any time if he or she so wishes.

6) DATA HANDLING

All data for this study is collected from patient files and hospitals' electronic patient registry (Artux, BCB Medical, Finland). All data is stored and secured in a specific electronic study subject register. Patient data is accessible to investigators only.

7) ASSESSMENTS AND OUTCOME MEASURES

1) The recurrence of instability (re-dislocation, subluxation, positive apprehension) is used as a primary outcome measure together with WOSI score two and five years postoperatively. Secondary outcome measures include: level and intensity to perform sports activities, subjective visual analogue estimation of the shoulder condition, Constant score, Oxford score, and SSV.
2) The pre-operative X-ray and MRA findings are compared with pre-operative CT imaging. The sensitivity and specificity to detect bony pathology in X-ray and MRA imaging is evaluated. All the acquired images are analysed by two separate radiologists at Turku University Hospital. The correlation of bone pathology detected in CT imaging with treatment outcome is further analysed.
3) The degenerative changes on x-rays two and five years post-operatively are graded according to Samilson and Prieto. APPENDIX 8. The differences between and within the study groups are analysed and compared with clinical findings and subjective patient satisfaction. All the acquired images are analysed by two separate radiologists at Turku University Hospital.

8) STATISTICS

The data will be analyzed using methods suitable for clinical trials regarding comparison of parallel treatment groups with repeated measurements. The primary technique will be the analysis of variance of repeated measurements together with generalized linear mixed models for longitudinal data and Cox regression models for survival data. These analytical tools cover methods for analysing different kinds of outcome variables, and are applicable although there is missing data in the measurements during the follow-up. The primary statistical software, to be used is the latest release of SAS system for windows, SAS Institute Inc., Cary, NC, USA.

9) SAMPLE SIZE AND POWER ANALYSES

The amount of residual instability after the operative stabilization is estimated to be 10% in the Latarjet group and 35% in the Bankart group. 90% of all redislocations are expected to occur within the first two years. This makes the possible number of redislocators 4 in the Latarjet group and 14 in the Bankart group at two years follow-up. If the mean WOSI score difference is 10 between the groups (estimated SD 17) and if α is set at 0.05 and power (1-β) at 80 %, a total of 47 patients per group is needed. To have enough statistical power in subgroup analysis, and to compensate for the possible drop-outs (estimate 15 %), a total of 120 patients are recruited into the study.

10) STUDY REGISTRATION

Study is registered in an electronic registry of medical studies at the website ClinicalTrials.gov.

11) AMENDMENT TO THE PROTOCOL

No deviation should be made from the protocol without an amendment. An amendment must be agreed to in writing and signed by the investigator and approved by the ethics committee before its implementation. An amendment, if it is of administrative nature only without affecting the treatment of the patients, may be implemented without ethics committee approval, but the investigator must submit the amendment to the ethics committee for information.

12) PUBLICATION OF THE RESULTS

The study groups are coded, and only after the one-year follow-up is completed for all patients the results are analyzed, the blinding is decoded, and a manuscript is prepared for publication. The investigators are free and willing to publish the results of this study in peer-reviewed journals. The results of this investigation are also published as part of MD Sami Elamo's thesis on operative treatment of anteroinferior shoulder instability.

13) STUDY CENTERS AND COLLABORATORS

list of collaborators	task
principal investigator:	
MD PhD Ville Äärimaa<br>Department of Orthopaedics and Traumatology<br>Turku University Hospital<br>P.O. Box 28, FIN-20701 Turku, Finland<br>Tel. +358 (0)2 313 0000<br>email: ville.aarimaa@utu.fi	co-ordination, analysing and reporting of results
study nurse:<br>Nurse Sanna Johansson<br>Department of Orthopaedics and Traumatology<br>Turku University Hospital<br>P.O. Box 28, FIN-20701 Turku, Finland<br>Tel. +358 (0)2 313 6132<br>email: sanna.johansson@tyks.fi	co-ordination
clinical researcher and PhD student:	
MD M.Sc. (Tech) Sami Elamo<br>Department of Orthopaedics and Traumatology<br>Turku University Hospital<br>P.O. Box 28, FIN-20701 Turku, Finland<br>Tel. +358 (0)2 313 0000<br>email: sami.elamo@utu.fi	clinical patient handling, analysing and reporting of results
Assisting clinical researchers:<br>MD PhD Tapio Flinkkilä<br>Department of Orthopaedics and Traumatology<br>Oulu University Hospital<br>P.O. Box 21<br>FIN-90029 OYS, Oulu, Finland<br>Tel. +358 8 3152011<br>email: tapio.flinkkila@ppshp.fi	clinical patient handling, analysing and reporting of results
MD PhD Antti Joukainen<br>Department of Orthopaedics and Traumatology<br>Kuopio University Hospital<br>P.O. Box 1777, FIN-70211 Kuopio, Finland	clinical patient handling, analysing and reporting of results

Tel. +358 17 173311
email: antti.joukainen@kuh.fi

MD Juha Kukkonen
Department of Orthopaedics and Traumatology
Satakunta Central Hospital
P.O. Box 28, FIN-28500 Pori, Finland
Tel. +358 (0)2 62 771
email: jupeku@utu.fi

clinical patient handling, analysing and reporting of results

MD PhD Janne Lehtinen
Hatanpää Hospital, Orthopaedics
P.O. Box 437, FIN-33101 Tampere, Finland
Tel. +358 3 565713
email: janne.lehtinen@tampere.fi

clinical patient handling, analysing and reporting of results

MD PhD Mika Paavola
Department of Orthopaedics and Traumatology
Töölön sairaala
Helsinki University Hospital
P.O.Box 266, FIN-00029 HUS, Helsinki, Finland
Tel. +358 9 471 87383
email: mika.paavola@hus.fi

clinical patient handling, analysing and reporting of results

MD PhD Vesa Lepola
Tampere University Hospital
P.O.Box 2000, FIN-33521 Tampere, Finland
Tel. +358 3 311611
email: vesa.lepola@pshp.fi

clinical patient handling, analysing and reporting of results

MD PhD Juha Paloneva
Central Finland Hospital
Keskussairaalantie 19, FIN-40620 Jyväskylä, Finland
Tel. +358 14 2693119
email: juha.paloneva@ksshp.fi

clinical patient handling, analysing and reporting of results

MD PhD Esa Tuominen
Department of Radiology
Turku University Hospital
P.O. Box 28, FIN-20701 Turku, Finland
Tel. +358 (0)2 313 0000
email: esa.tuominen@tyks.fi

MRI analyses, analysing and reporting of results

14) PICOS

P	Patients	120 young (16-25 year old) male patients with clinically documented anteroinferior shoulder instability requiring operative intervention.
I	Intervention	Open Latarjet procedure (n=60)
C	Comparator	Arthroscopic Bankart procedure (n=60)
O	Outcome	Primary outcome measures: recurrence of instability (re-dislocation, subluxation, positive apprehension) and WOSI score.
		Secondary outcome measures: level and intensity to perform sports activities, subjective visual analogue estimation of the shoulder condition, Constant score, Oxford score, and SSV.
S	Study design	Randomized controlled study with parallel treatment groups.

Kukkonen J, *et al. Br J Sports Med* 2022; 56:327–332. doi: 10.1136/bjsports-2021-104028

15) FLOWCHART

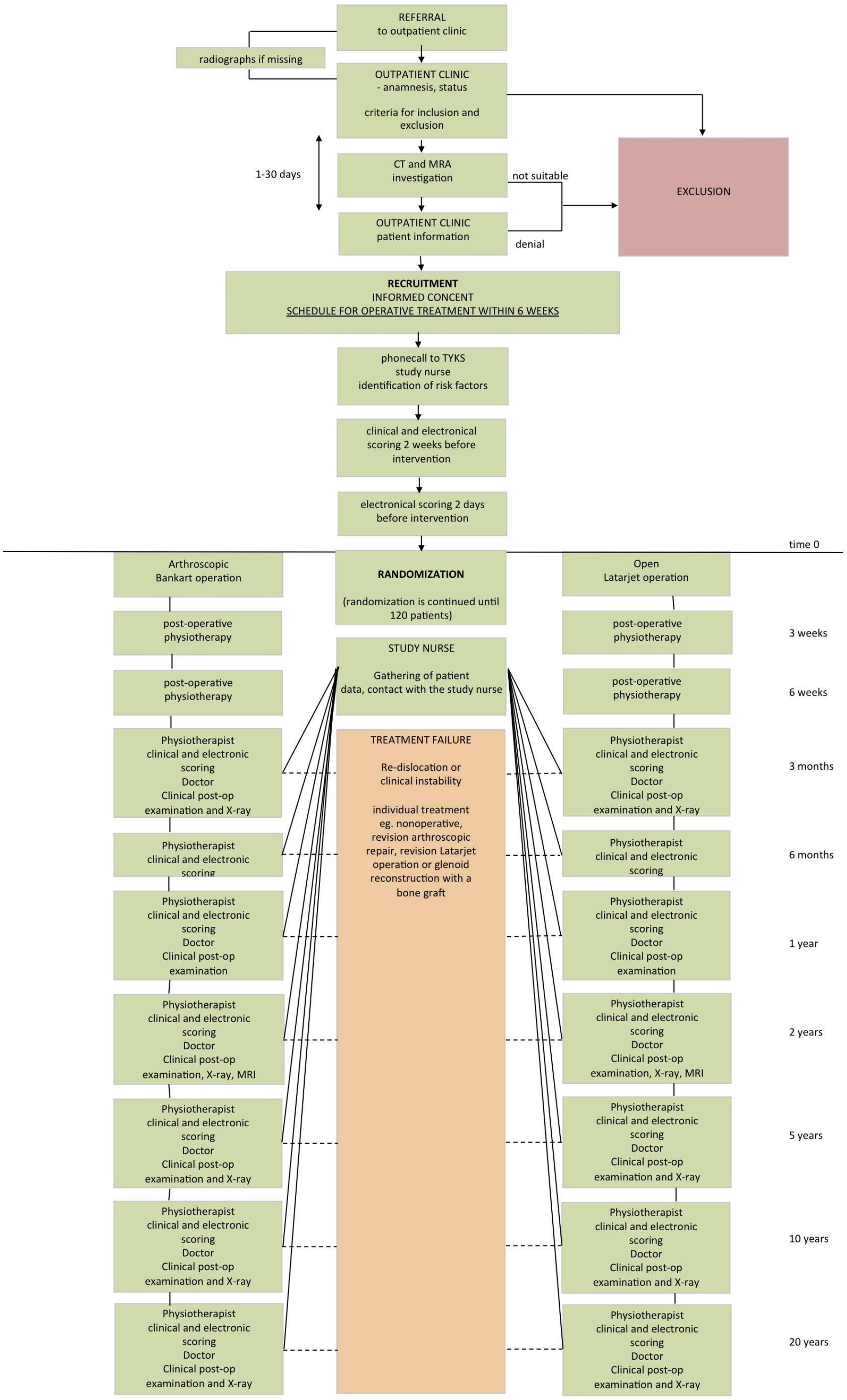

Kukkonen J, *et al. Br J Sports Med* 2022; 56:327–332. doi: 10.1136/bjsports-2021-104028

16) TIMETABLE

The study protocol is submitted the Ethics Committee and after the approval the recruitment of participants is commenced. The estimated time of recruitment is two years until the end of year 2015. Thereafter the follow-up will take up to 5 years and the study will be completed by year 2021.

17) FUNDING

At the beginning all participating hospitals will take care of their own patients, imaging and treatment expenses as part of normal patient care. The study nurse and the PhD student are partially employed by the principal investigator and are at use for all clinical researchers for the purpose of this study. Extra funding is applied for the follow-up imaging studies and other cost relating to this study.

18) REFERENCES

Ahmed I, Ashton F, Robinson CM
Arthroscopic Bankart Repair and Capsular Shift for Recurrent Anterior Shoulder Instability: Functional Outcomes and Identification of Risk Factors for Recurrence.
J Bone Joint Surg (am) 2012; 94-A: 1308-1315.

Allain J, Goutallier D, Glorion C
Long-term results of the Latarjet procedure for the treatment of anterior instability of the shoulder.
J Bone Joint Surg (am) 1998; 80: 841-52.

Auffarth A, Schauer J, Matis N, Kofler B, Hitzl W, Resch H
The J-bone graft for anatomical glenoid reconstruction in recurrent posttraumatic anterior shoulder dislocation.
Am J Sports Med. 2008; 36: 638-47.

Balg F, Boileau P
The instability severity index score. A simple pre-operative score to select patients for arthroscopic or open shoulder stabilisation.
J Bone Joint Surg (Br) 2007; 89: 1470-7.

Boileau P, Villalba M, Hery J-Y, Balg F, Ahrens P, Neyton L
Risk Factors for Recurrence of Shoulder Instability After Arthroscopic Bankart Repair.
J Bone Joint Surg 2006; 88-A:1755–1763.

Boileau P, O'Shea K, Vargas P, Pinedo M, Old J, Zumstein M
Anatomical and Functional Results After Arthroscopic Hill-Sachs Remplissage.
J Bone Joint Surg (am) 94-A:618-626.

Castagna A, Markopoulos N, Conti M, Rose GD, Papadakou E, Garofalo R. Arthroscopic bankart suture-anchor repair: radiological and clinical outcome at minimum 10 years of follow-up.
Am J Sports Med 2010; 38:2012-6.

Castagna A, Rose GD, Borroni M, Cillis BD, Conti M, Garofalo R, Ferguson D, Portinaro N, Arthroscopic stabilization of the shoulder in adolescent athletes participating in overhead or contact sports.
Arthroscopy 2012: 28:309-15.

Cho SH, Cho NS, Rhee YG
Preoperative analysis of the Hill-Sachslesion in anterior shoulder instability: how to predict engagement of the lesion.
Am J Sports Med. 2011;39;2389-2395.

Constant CR, Murley AH
A clinical method of functional assessment of the shoulder.
Clin Orthop 1987; 214:160-4

de Beer JF. Roberts C
Glenoid bone defects--open latarjet with congruent arc modification.
Orthop Clinics North Am 2010;41:407-15.

Flinkkila T. Hyvonen P. Ohtonen P. Leppilahti J
Arthroscopic Bankart repair: results and risk factors of recurrence of instability.
Knee Surg Sports Traumatol, Arthrosc 2010;18:1752-8.

Gilbart MK, Gerber C
Comparison of the subjective shoulder value and the Constant score.
J Shoulder Elbow Surg 2007; 16: 717-721.

Liavaag S, Svenningsen S, Reikeras O
The epidemiology of shoulder dislocations in Oslo.
Scand J Med Sci Sports 2011;21:e334-40.

Hobby J, Griffin D, Dunbar M, Boileau P
Is arthroscopic surgery for stabilisation of chronic shoulder instability as effective as open surgery?: A systematic review and meta-analysis of 62 studies including 3044 arthroscopic operations.

J Bone Joint Surg (br) 2007;89-B:1188-1196.

Hovelius L, Augustini BG, Fredin H, Johansson O, Norlin R, Thorling J
Primary anterior dislocation oft he shoulder in young patients. A ten-year prospective study.
J Bone Joint Surg Am 1996;78:1677-84.

Hovelius L, Sandstrom B, Sundgren K, Saebo M
One hundred eighteen Bristow-Latarjet repairs for recurrent anterior dislocation of the shoulder prospectively followed for fifteen years: study I--clinical results.
J Shoulder Elbow Surg 2004; 13: 509-16.

Hovelius L, Olofsson A, Sandstrom B
Nonoperative treatment of primary anterior shoulder dislocation in patients forty years of age and younger. A prospective twenty-five-year follow-up.
J Bone Joint Surg Am 2008;90:945-52.

Hovelius L, Vikerfors O, Olofsson A, Svensson O, Rahme H
Bristow-Latarjet and Bankart: a comparative study of shoulder stabilization in 185 shoulders during a seventeen-year follow-up.
J Shoulder Elbow Surg 2011;20:1095-101.

Hovelius L, Sandstrom B, Olofsson A, Svensson O, Rahme H
The effect of capsular repair, bone block healing, and position on the results of the Bristow-Latarjet procedure (study III): long-term follow-up in 319 shoulders.
J Shoulder Elbow Surg 2012;21:647-60

Hudak PL, Amadio PC, Bombardier C
Development of an upper extremity outcome measure: the DASH (disabilities of the arm, shoulder and hand). The Upper Extremity Collaborative Group (UECG).
Am J Ind Med. 1996;29:602–608.

Kaar SG, Fening SD, Jones MH, Colbrunn RW, Miniaci A. Effect of humeral head defect size on glenohumeral stability: a cadaveric study of simulated Hill-Sachs defects.
Am J Sports Med. 2010;38:594-599.

Kirkley A, Griffin S, McLintock H, Ng L
The Development and evaluation of a disease specific quality of life measurement tool for shoulder instability. The Western Ontario Shoulder Instability Index (WOSI).
Am J Sports Med 1998;26:764-72.

Kodali P, Jones MH, Polster J, Miniaci A
Fening SD. Accuracy of measurement of Hill-Sachs lesions with computed tomography.
J Shoulder Elbow Surg 2011; 20:1328-34.

Matthes G, Horvath V, Seifert J, ym. Oldie but goldie: Bristow-Latarjet procedure for anterior shoulder instability.
J Orthop Surg (Hong Kong) 2007;15:4-8.

Park MJ, Tjoumakaris FP, Garcia G, Patel A, Kelly JD 4th
Arthroscopic remplissage with Bankart repair for the treatment of glenohumeral instability with Hill-Sachs defects.
Arthroscopy 2011;27:1187-94.

Phatia S, McGill KC, Ghodadra NS, Baudi P, Romeo AA, Provencher MT
Radiographic and arthroscopic evaluation of glenoid and humeral head bone loss.
Shoulder Instability. A comprehensive approach. Elsevier, Philadelphia 2012 p. 170 - 185.

Porcellini G, Campi F, Pegreffi F, Castagna A, Paladini P
Predisposing factors for recurrent shoulder dislocation after arthroscopic treatment.
J Bone Joint Surg (am) 2009;91:2537-42.

Privitera DM, Bisson LJ, Marzo JM
Minimum 10-year follow-up of arthroscopic intra-articular Bankart repair using bioabsorbable tacks.
Am J Sports Med 2012;40:100-7.

Roberts SN, Taylor DE, Brown JN, Hayes MG, Saies A
Open and arthroscopic techniques for the treatment of traumatic anterior shoulder instability in Australian rules football players.
J Shoulder Elbow Surg 1999; 8: 403-9.

Rockwood CA, Matsen FA III, Wirth MA, Lippitt SB
The Shoulder. 4Th Ed., Saunders 2009

Samilson RL, Prieto V
Dislocation arthropathy of the shoulder.
J Bone Joint Surg Am 1983; 65(4): 456-460.

Soderlund T, Mattila VM, Visuri TI, Pihlajamaki HK
Long-term outcome of a transglenoid suture technique for anterior shoulder instability in young adults.
J Bone Joint Surg (Br) 2008;90:189-93.

van der Linde JA, van Kampen DA, Terwee CB, Dijksman LM, Kleinjan G, Willems WJ
Long-term results after arthroscopic shoulder stabilization using suture anchors: an 8- to 10-year follow-up.
Am J Sports Med 2011;39:2396-403.

Voos JE, Livermore RW, Feeley BT, Altchek DW, Williams RJ, Warren RF, Cordasco FA, Allen AA
Prospective evaluation of arthroscopic bankart repairs for anterior instability.
Am J Sports Medicine 2010;38:302-7.

Wellmann M, Petersen W, Zantop T, Herbort M, Kobbe P, Raschke, MJ, Hurschler C
Open Shoulder Repair of Osseous Glenoid Defects: Biomechanical Effectiveness of the Latarjet Procedure Versus a Contoured Structural Bone Graft.
Am J Sports Med 2009;37:87-94.

Wellmann M, de Ferrari H, Smith T, Petersen W, Siebert CH, Agneskirchner JD, Hurschler C
Biomechanical investigation of the stabilization principle of the Latarjet procedure.
Arch Orthop Trauma Surg 2012; 132: 377-86.

Yamamoto N, Muraki T, Sperling JW, Steinmann SP, Cofield RH, Itoi E, An KN
Stabilizing mechanism in bone-grafting of a large glenoid defect.
J Bone Joint Surg (am) 2010; 92: 2059-66.

Young AA, Walch G
Open bony augmentation of glenoid bone loss. The Latarjet and variants- surgical technique. In: Provencher MT, Romeo AA eds. p. 405 -416.
Shoulder Instability. A comprehensive approach. Elsevier, Philadelphia 2012 p. 197 – 208

Zhu YM, Lu Y, Zhang J, Shen JW, Jiang CY
Arthroscopic Bankart repair combined with remplissage technique for the treatment of anterior shoulder instability with engaging Hill-Sachs lesion: a report of 49 cases with a minimum 2-year follow-up.
Am J Sports Med 2011: 39: 1640-7.

APPENDIXES

APPENDIX 1

1) The glenoid bone defect is considered significant when the tangential length of the defect is equal or more than 50% of the maximal width of the glenoid surface on a two dimensional en face CT view (Gerber 2002).

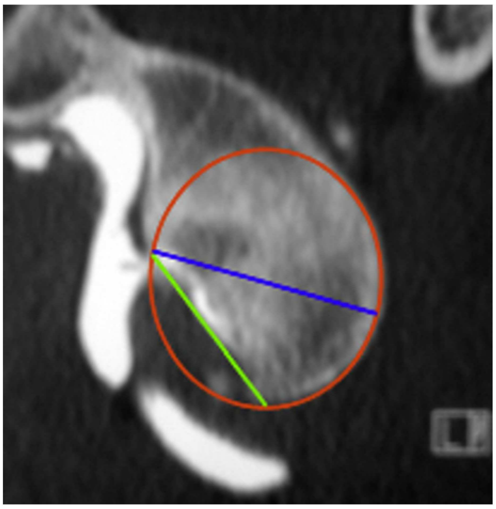

2) The humeral defect is considered significant when the maximal width of the Hill-Sachs defect is equal or more than 40% of the diameter of the humeral head on a two dimensional axial CT view (Cho 2011, Kaar 2010).

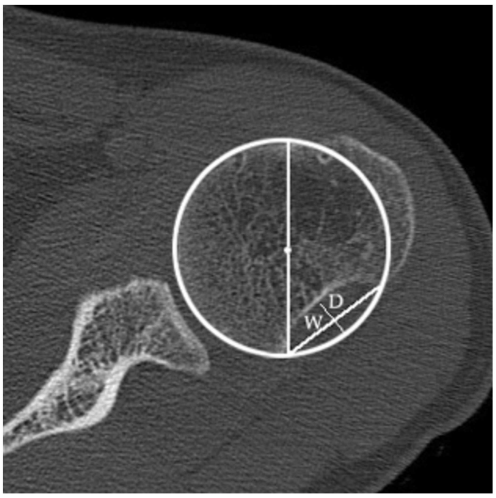

Kukkonen J, *et al. Br J Sports Med* 2022; 56:327–332. doi: 10.1136/bjsports-2021-104028

APPENDIX 2

Constant score

OUT-PATIENT CLINIC **SHOULDER UNIT**

CONSTANT SCORE

Patient's Details

Operation/Diagnosis: **Date:**__________

Side: **R** **L**

Examination: Pre-op
3 months 6months
1 year 2 years ___ years

A.- Pain (/15): **Average (1 + 2)** ☐ **A**

1. Do you have pain in your shoulder (normal activities)?
No =15 pts, Mild pain = 10 pts, Moderate = 5 pts, Severe or permanent = 0. ____

2. Linear scale:
If "0" means no pain and "15" is the maximum pain you can experience, please circle where is the level of pain of your shoulder. (Points given are inverse to the scale. E.g. level 5 in the scale means 10 points)

**Level of pain:**	0	1	2	3	4	5	6	7	8	9	10	11	12	13	14	15
Points:	15	14	13	12	11	10	9	8	7	6	5	4	3	2	1	0

____

B.- Activities of daily living (/20) **Total (1 + 2 + 3 + 4)** ☐ **B**

1. Is your occupation or daily living limited by your shoulder?
No = 4, Moderate limitation = 2, Severe limitation = 0 ____

2. Are your leisure and recreational activities limited by your shoulder?
No = 4, Moderate limitation = 2, Severe limitation = 0 ____

3. Is your night sleep disturbed by your shoulder?
No = 2, Sometimes = 1, Yes = 0 ____

4. State to what level you can use your arm for painless, reasonably activities.
Waist = 2, Xiphoid (sternum) = 4, Neck = 6, Head = 8, Above head = 10 ____

C.- Range of movement (leave this for the doctor or physiotherapist) **(/40):** **Total (1 + 2 + 3 + 4)** ☐ **C**

1.- FWD Flexion: ____
0 - 30 0 pts
31 -60 2 pts
61 - 90 4 pts
91 - 120 6 pts
121- 150 8 pts
> 150 10 pts

2.- Abduction: ____
0 - 30
31 - 60
61 - 90
91 - 120
121 - 150
> 150

3.- External Rotation: ____
Hand behind head & elbow forward 2
Hand behind head & elbow back 4
Hand above head & elbow forward 6
Hand above head & elbow back 8
Full elevation of arm 10

4.- Internal Rotation: (Dorsum hand to) ____
Thigh 0
Buttock 2
SI joint 4
Waist 6
T12 8
Between shoulder blades 10

D.- Power (/25): Points: average (kg) x 2 = ☐ **D**
First pull: Second pull: Third pull: Fourth pull: Fifth pull:
Average pulls:

TOTAL (/100): A + B + C+ D ☐

APPENDIX 3

WOSI Western Ontario Shoulder Instability Index

SECTION A:
Physical Symptoms

INSTRUCTIONS TO PATIENTS

The following questions concern the physical symptoms you have experienced due to your shoulder problem. In all cases, please enter the amount of the symptom you have experienced in the last week. (Please answer with an "X" on the horizontal line.)

1. How much pain do you experience in your shoulder with overhead activities?
   no pain ——— extreme pain
2. How much aching or throbbing do you experience in your shoulder?
   no aching/throbbing ——— extreme aching/throbbing
3. How much weakness or lack of strength do you experience in your shoulder?
   no weakness ——— extreme weakness
4. How much fatigue or lack of stamina do you experience in your shoulder?
   no fatigue ——— extreme fatigue
5. How much clicking, cracking or snapping do you experience in your shoulder?
   no clicking ——— extreme clicking
6. How much stiffness do you experience in your shoulder?
   no stiffness ——— extreme stiffness
7. How much discomfort do you experience in your neck muscles as a result of your shoulder?
   no discomfort ——— extreme discomfort
8. How much feeling of instability or looseness do you experience in your shoulder?
   no instability ——— extreme instability
9. How much do you compensate for your shoulder with other muscles?
   not at all ——— extreme
10. How much loss of range of motion do you have in your shoulder?
    no loss ——— extreme loss

‡ On the actual form the lines are 100-mm long. This form is reproduced by permission of the Fowler · Kennedy Sports Medicine Clinic.

SECTION B:
Sports/Recreation/Work

INSTRUCTIONS TO PATIENTS

The following section concerns how your shoulder problem has affected your work, sports or recreational activities in the past week. For each question, please indicate the amount with an "X" on the horizontal line.

11. How much has your shoulder limited the amount you can participate in sports or recreational activities?
    not limited ——— extremely limited
12. How much has your shoulder affected your ability to perform the specific skills required for your sport or work? (If your shoulder affects both sports and work, consider the area that is most affected.)
    not affected ——— extremely affected
13. How much do you feel the need to protect your arm during activities?
    not at all ——— extreme
14. How much difficulty do you experience lifting heavy objects below shoulder level?
    no difficulty ——— extreme difficulty

SECTION C:
Lifestyle

INSTRUCTIONS TO PATIENTS

The following section concerns the amount that your shoulder problem has affected or changed your lifestyle. Again, please indicate the appropriate amount for the past week with an "X" on the horizontal line.

15. How much fear do you have of falling on your shoulder?
    no fear ——— extreme fear
16. How much difficulty do you experience maintaining your desired level of fitness?
    no difficulty ——— extreme difficulty
17. How much difficulty do you have "roughhousing or horsing around" with family or friends?
    no difficulty ——— extreme difficulty
18. How much difficulty do you have sleeping because of your shoulder?
    no difficulty ——— extreme difficulty

SECTION D:
Emotions

INSTRUCTIONS TO PATIENTS

The following questions relate to how you have felt in the past week with regard to your shoulder problem. Please indicate your answer with an "X" on the horizontal line.

19. How conscious are you of your shoulder?
    not conscious ——— extremely conscious
20. How concerned are you about your shoulder becoming worse?
    no concern ——— extremely concerned
21. How much frustration do you feel because of your shoulder?
    no frustration ——— extremely frustrated

Kukkonen J, *et al. Br J Sports Med* 2022; 56:327–332. doi: 10.1136/bjsports-2021-104028

APPENDIX 4

Oxford Instability Score

OXFORD SHOULDER INSTABILITY SCORE

Problems with your shoulder

RIGHT ☐
LEFT ☐

✓ tick **one** box for each question

1 During the last **6 months** ...
how many times has your shoulder slipped out of joint (or dislocated)?

Not at all in 6 months	1 or 2 times in 6 months	1 or 2 times per month	1 or 2 times per week	More often than 1 or 2 times/week
☐	☐	☐	☐	☐

2 During the last **3 months** ...
have you had any trouble (or worry) with putting on a T-shirt or pullover *because of your shoulder*?

No trouble/ no worries	Slight trouble or worry	Moderate trouble or worry	Extreme difficulty	Impossible to do
☐	☐	☐	☐	☐

3 During the last **3 months** ...
how would you describe the <u>worst</u> pain you have had *from your shoulder*?

None	Mild ache	Moderate	Severe	Unbearable
☐	☐	☐	☐	☐

4 During the last **3 months** ...
how much has *the problem with your shoulder* interfered with your usual work? (including school or college work, or housework)

Not at all	A little bit	Moderately	Greatly	Totally
☐	☐	☐	☐	☐

5 During the last **3 months** ...
have you avoided any activities due to *worry about your shoulder* – feared that it might slip out of joint?

No, not at all	Very occasionally	Some days	Most days or more than one activity	Every day or many activities
☐	☐	☐	☐	☐

6 During the last **3 months** ...
has *the problem with your shoulder* prevented you from doing things that are important to you?

No, not at all	Very occasionally	Some days	Most days or more than one activity	Every day or many activities
☐	☐	☐	☐	☐

Oxford Instability Shoulder Score

7 During the last **3 months** ...
how much has *the problem with your shoulder* interfered with your social life? (including sexual activity – if applicable)

Not at all	Occasionally	Some days	Most days	Every day
☐	☐	☐	☐	☐

8 During the last **4 weeks** ...
how much has *the problem with your shoulder* interfered with your sporting activities or hobbies?

Not at all	A little/ occasionally	Some of the time	Most of the time	All of the time
☐	☐	☐	☐	☐

9 During the last **4 weeks** ...
how often has your shoulder been 'on your mind' – how often have you thought about it?

Never, or only if someone asks	Occasionally	Some days	Most days	Every day
☐	☐	☐	☐	☐

10 During the last **4 weeks** ...
how much has *the problem with your shoulder* interfered with your ability – or willingness – to lift heavy objects?

Not at all	Occasionally	Some days	Most days	Every day
☐	☐	☐	☐	☐

11 During the last **4 weeks** ...
how would you describe the pain you *usually* had from your shoulder?

None	Very mild	Mild	Moderate	Severe
☐	☐	☐	☐	☐

12 During the last **4 weeks** ...
have you avoided lying in certain positions, in bed at night, *because of your shoulder*?

No nights	Only 1 or 2 nights	Some nights	Most nights	Every night
☐	☐	☐	☐	☐

Nuffield Department of Orthopaedic Surgery

Nuffield NHS Orthopaedic Centre NHS Trust

Kukkonen J, *et al. Br J Sports Med* 2022; 56:327–332. doi: 10.1136/bjsports-2021-104028

APPENDIX 5

ISIS score

**Prognostic factors**	**Points**
Age at Surgery (yrs)	
≤ 20	2
> 20	0
Degree of sport participation (pre-operative)	
Competitive	2
Recreational or none	0
Type of Sport (pre-operative)	
Contact or forced overhead	1
Other	0
Shoulder Hyperlaxity*	
Hyperlaxity (anterior/inferior)	1
Normal	0
Hill-Sachs lesion on AP radiograph	
Visible on external rotation	2
Not visible on external rotation	0
Glenoid loss of contour on AP radiograph	
Loss of contour	2
No lesion	0
**Total (points)**	**10**

Kukkonen J, *et al. Br J Sports Med* 2022; 56:327–332. doi: 10.1136/bjsports-2021-104028

APPENDIX 6

Required X-ray projections

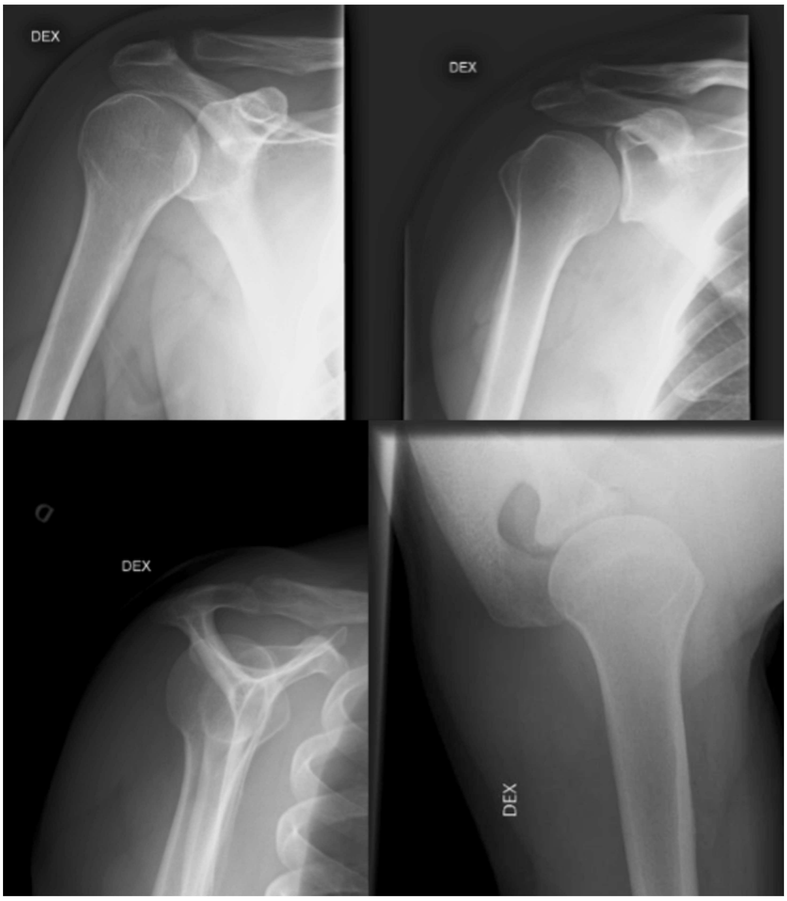

Kukkonen J, *et al. Br J Sports Med* 2022; 56:327–332. doi: 10.1136/bjsports-2021-104028

APPENDIX 7

<u>preoperative eligibility criteria include:</u> asessement of clinical glenohumeral stability (apprehension and relocation tests, jerk test, clinical signs of hyperlaxity (positive sulcus sign or Gagey test)), and inclusion and exclusion criteria

<u>clinical scoring includes</u>: Constant score and pre- or postoperative anamnestic characteristics.

<u>1) preoperative anamnestic characteristics include</u>: age at primary injury, mechanism of primary injury, number of dislocation episodes, degree of instability (dislocations at sleep, during daily activities, at sports, no dislocations), level (elite, competitive, recreational, none) and intensity (times per week) to perform sports activities (type of sport), pain at rest, subjective visual analogue estimation of the shoulder condition, smoking habits, other diseases, type and ability to work, handedness

2) <u>postoperative anamnestic characteristic include</u>: number of dislocation episodes, degree of instability (dislocations at sleep, during daily activities, at sports, no dislocations), level and intensity to perform sports activities, pain at rest, subjective visual analogue estimation of the shoulder condition, and ability to work, and subjective satisfaction on the treatment outcome on a 5-point Likert scale.

<u>electronic scoring includes</u>: Western Ontario Shoulder Instability index (WOSI), Oxford Instability Score (Oxford score), Subjective Shoulder Value (SSV)

<u>clinical postoperative examination includes</u>: clinical glenohumeral stability (apprehension and relocation tests).

D= doctor
P= physiotherapist
E= electronic

Assessment	recruitment	2 weeks preop	randomization	2d preop	operation	3 wks postop	6 wks postop	3 mts postop	6 mts postop	1yr postop	2yr postop	5yr postop	10yr postop	20yr postop
Preop eligib. criteria	D													
Clinical scoring		P						P	P	P	P	P	P	P
Electronic scoring		E		E				E	E	E	E	E	E	E
Postopphysiotherapy						P	P							
Clin postop exam								D		D	D	D	D	D
x-ray	D							D			D	D	D	D
CT	D													
MRA	D										D			

Kukkonen J, *et al. Br J Sports Med* 2022; 56:327–332. doi: 10.1136/bjsports-2021-104028

APPENDIX 8

Classification of osteoarthritic changes by Samilson et Prieto.

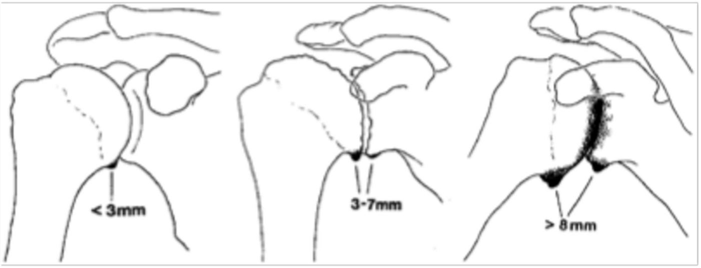

a b c

Fig. 60. Radiological classification of dislocation arthropathy. a Mild arthrosis evidence on the anteroposterior radiograph of an inferior humeral or glenoid exostosis, or both, measuring <3 mm. b Moderate arthrosis evidence on the anteroposterior radiograph of an inferior humeral or glenoid exostosis, or both, measuring between 3 and 7 mm, with slight glenohumeral-joint irregularity. c Severe arthrosis evidence on the anteroposterior radiograph of an inferior humeral or glenoid osteosis, or both, measuring >8 mm, with glenohumeral narrowing and sclerosis. (From [117])

Kukkonen J, *et al. Br J Sports Med* 2022; 56:327–332. doi: 10.1136/bjsports-2021-104028
